# Isolation and Characterization of Canine Amniotic Membrane-Derived Multipotent Stem Cells

**DOI:** 10.1371/journal.pone.0044693

**Published:** 2012-09-14

**Authors:** Sang-Bum Park, Min-Soo Seo, Hyung-Sik Kim, Kyung-Sun Kang

**Affiliations:** 1 Adult Stem Cell Research Center, College of Veterinary Medicine, Seoul National University, Seoul, Republic of Korea; 2 Laboratory of Stem Cell and Tumor Biology, Department of Veterinary Public Health, College of Veterinary Medicine, Seoul National University, Seoul, Republic of Korea; 3 BK 21 Program for Veterinary Sciences, College of Veterinary Medicine, Seoul National University, Seoul, Republic of Korea; Bauer Research Foundation, United States of America

## Abstract

Recent studies have shown that amniotic membrane tissue is a rich source of stem cells in humans. In clinical applications, the amniotic membrane tissue had therapeutic effects on wound healing and corneal surface reconstruction. Here, we successfully isolated and identified multipotent stem cells (MSCs) from canine amniotic membrane tissue. We cultured the canine amniotic membrane-derived multipotent stem cells (cAM-MSCs) in low glucose DMEM medium. cAM-MSCs have a fibroblast-like shape and adhere to tissue culture plastic. We characterized the immunophenotype of cAM-MSCs by flow cytometry and measured cell proliferation by the cumulative population doubling level (CPDL). We performed differentiation studies for the detection of trilineage multipotent ability, under the appropriate culture conditions. Taken together, our results show that cAM-MSCs could be a rich source of stem cells in dogs. Furthermore, cAM-MSCs may be useful as a cell therapy application for veterinary regenerative medicine.

## Introduction

Recently, the study of stem cells has focused on the therapeutic effects of the cells. Stem cells have a multipotent differentiation capacity and self-renewal ability. Initially, multipotent stem cells (MSCs) were isolated and characterized from bone marrow [1]. The current sources of MSCs are from various tissues such as umbilical cord blood, adipose tissue, amniotic fluid and peripheral blood [2]–[6]. The placenta has an important role in the development and survival of the fetus, supplying nutrients and oxygen. The placenta has three-layer structure including the amnion, chorion and deciduas [7]. Commonly, after parturition, the placenta is treated as medical waste and discarded. However, amniotic membranes have the potential to be used as a clinical application for wound healing and cornea surface reconstruction [7]. In humans, there are reports of MSCs derived from placenta, especially, from the amniotic membrane [8], [9].

Additionally, we demonstrated that cAM-MSCs have a multilineage differentiation capacity and self-renewal ability. Our results showed that the typical morphology of cAM-MSCs is similar to that of human MSCs and that, the cells displayed vigorous cell proliferation. In the differentiation studies, cAM-MSCs showed adipogenesis, osteogenesis, neurogenesis and chondrogenesis ability *in vitro*. Therefore, we suggest that cAM-MSCs may represent a rich source of stem cells that may be useful in veterinary medicine. Furthermore, cAM-MSCs could be used therapeutically in canine regenerative medicine studies.

## Materials and Methods

Although we use animal tissue, harvesting of tissues did not involve any invasive or inhumane methodology. In fact, sacrificing live animal for harvesting was not needed at all. We used amniotic membranes that are normally discarded after they are separated by cesarean sectioning for deliveries in our department (Seoul National University Hospital for Animal). The cesarean sections were carried out for non-research purpose at approximately 60–69 days of gestation by qualified veterinarians. They were provided free of charge by the department for the purposes of the study. Such isolated membranes were only used in isolating and characterizing stem cells from the tissue. All safety compliances were strictly observed and adhered to the Policy and Regulation for Care and Use of Laboratory Animals (Institute of Laboratory Animal Resources Seoul National University).

### Animals

Healthy adult mixed-breed dogs (n = 6; 4.5±0.4 kg) were used. Applicable institutional and governmental regulations concerning the ethical use of animals were followed during the course of this research. This investigation was performed in accordance with the guidelines of the “Guide for the Care and Use of Laboratory Animals” of Seoul National University. In the cesarean-section delivery, the dogs were pre-medicated with acepromazine maleate (0.1 mg/kg; Sedaject, Samwoo medical, Yesan, Korea) and then, thiopental sodium (15 mg/kg; Pentotal, Joongwei pharmaceutical, Seoul, Korea) was injected intravenously to induce anesthesia. Isoflurane (AErrane, Baxter, Mississauga, ON, Canada) was used to maintain anethesia. Under sterile conditions, the procedure was performed.

**Table 1:** List of PCR primers for differentiation markers.

Markers	Name	Sequence of primers	Size	Cycle	Temp. (°C)
	LPL	Forward : ACACATTCACAAGAGGGTCAC Reverse : CTCTGCAATCACACGGATG	132	32	60
Adipocyte	LEPTIN	Forward : CTATCTGTCCTGTGTTGAAGCTG Reverse : TGTGTGAAATGTCATTGATCCTG	102	32	60
	FABP4	Forward : ATCAGTGTAAACGGGGATGTG Reverse : GACTTTTCTGTCATCCGCAGTA	117	32	60
	SPARC	Forward : TGAGAAGGTATGCAGCAACG Reverse : AGTCCAGGTGGAGTTTGTGG	110	32	56
Osteocyte	MSX2	Forward : TCCGCCAGAAACAATACCTC Reverse : AAGGGTAGGACGCTCCGTAT	243	32	56
	COL1A1	Forward : CACCTCAGGAGAAGGCTCAC Reverse : ATGTTCTCGATCTGCTGGCT	124	32	56
	BGLAP	Forward : GTGGTGCAACCTTCGTGTC Reverse : GCTCGCATACTTCCCTCTTG	132	34	58
Neurocyte	GFAP	Forward : TCCGAGGGGGCAAAAGCACC Reverse : GGCAGGCTGCTAACCGAGAGC	104	30	62
	MAP2	Forward : CAGCGACAAGGCCGACACGT Reverse : GGGCCAAACTCGACACCCGG	336	34	66
Chondrocyte	COL2A1	Forward : GAAACTCTGCCACCCTGAATG Reverse : GCTCCACCAGTTCTTCTTGG	156	34	64
	AGGRECAN	Forward : ATCAACAGTGCTTACCAAGACA Reverse : ATAACCTCACAGCGATAGATCC	122	32	60
Housekeeping	GAPDH	Forward : AACATCATCCCTGCTTCCAC Reverse : TCCTTGGAGGCCATGTAGAC	392	24	58

### Cell Isolation and Culture

Cell isolation was performed as previously described with some modification [8], [10]. All placental samples were obtained through cesarean-section deliveries from canines. To separate the amniotic membrane from the whole placenta, the amniotic membrane was peeled off from the chorionic membrane mechanically. Under sterile conditions, the collected amniotic membranes were rinsed with normal saline (0.9%) several times. Generally, amniotic membrane (AM) composed of epithelial monolayer and an avascular stroma. The collected amniotic membranes were conducted with enzymatic and mechanical digestion treatment in 2 steps. Step 1; to avoid contamination of epithelial cells, in cell isolation of amniotic membrane, the collected AM was treated with trypsin-EDTA (0.25%) at 37°C for 30 minutes. After trypsin-EDTA treatment, the AM was washed with normal saline for 3∼4 times. Step 2; to isolate pure mesenchymal cells from canine AM without epithelial cells, the washed AM was chopped with a surgical blade. Thereafter, it was digested in collagenase type I (2 mg/ml; Worthington biochemical, Freehold, NJ) at 37°C for approximately 3∼4 hours. After enzyme digestion, washed in phosphate-buffered saline (PBS) (Cellgro, USA) by centrifugation at 350×*g* for 5 min. The cell pellet was resuspended in the basal culture medium, low glucose Dulbecco’s Modified Eagle’s Medium (LG-DMEM; Gibco BRL, USA) containing 10% FBS (Fetal bovine serum; Gibco BRL, USA). The cells were seeded into T75 polystyrene cell culture flasks (Nunc, USA) and incubated in a humidified atmosphere with 5% CO_2_. The basal culture medium was changed 3 times a week and passaged once the cells reached 80–90% confluency.

**Table 2:** List of quatitative RT-PCR primers for stem cell markers.

Name	Sequence of primers	Temp. (°C)
OCT4	Forward : TCGTGAAGCCGGACAAGGAGAAG Reverse : AGGAACATGTTCTCCAGGTTGCCT	60
SOX2	Forward : AACCCCAAGATGCACAACTC Reverse : CGGGGCCGGTATTTATAATC	60
NANOG	Forward : CCTGCATCCTTGCCAATGTC Reverse : TCCGGGCTGTCCTGAGTAAG	60
KLF4	Forward : CCATGGGCCAAACTACCCAC Reverse : TGGGGTCAACACCATTCCGT	60

### Cumulative Population Doubling Level Analysis

The proliferation and growth efficiency of cAM-MSCs were determined by the total cumulative population doubling level (CPDL) using the formula CPDL  =  ln (Nf/Ni ) ln2, where Ni is the initial seeding cell number, Nf is the final harvest cell numbers and ln is the natural log. The cells (5×10^4^) were plated in triplicate in a 6-well culture plate (Nunc) and subcultured 5–7 days later. The final cell numbers were counted and 5×10^4^ cells were re-plated. To yield the cumulated doubling level, the population doubling for each passage was calculated and then added to the population doubling levels of the previous passages.

### Flow Cytometry

To determine the immunophenotype of cAM-MSCs, the cells were stained with specific antibodies for FACS analysis, following the protocol provided by the supplier (BD Biosciences, USA). Briefly, the cAM-MSCs were trypsinized and washed several times with PBS. The suspended cells were aliquoted (approximately 1×10^6^ cells) for specific antibody staining. The cells were immunostained with the following antibodies: Mouse anti-human CD3, mouse anti-human CD11c, mouse anti-human CD28, mouse anti-human CD34, mouse anti-human CD38, mouse anti-human CD41a, mouse anti-human CD45, mouse anti-human CD62L, mouse anti-human CD90 (BD Biosciences) and mouse anti human CD105 (Serotec, USA). The antibodies were conjugated with Fluorescein isothiocyanate (FITC) or phycoerythrin (PE). Analysis was determined by the use of FACS Calibur™ (BD Biosciences) and Cell Quest Pro™ (BD Biosciences) software.

**Figure 1:**
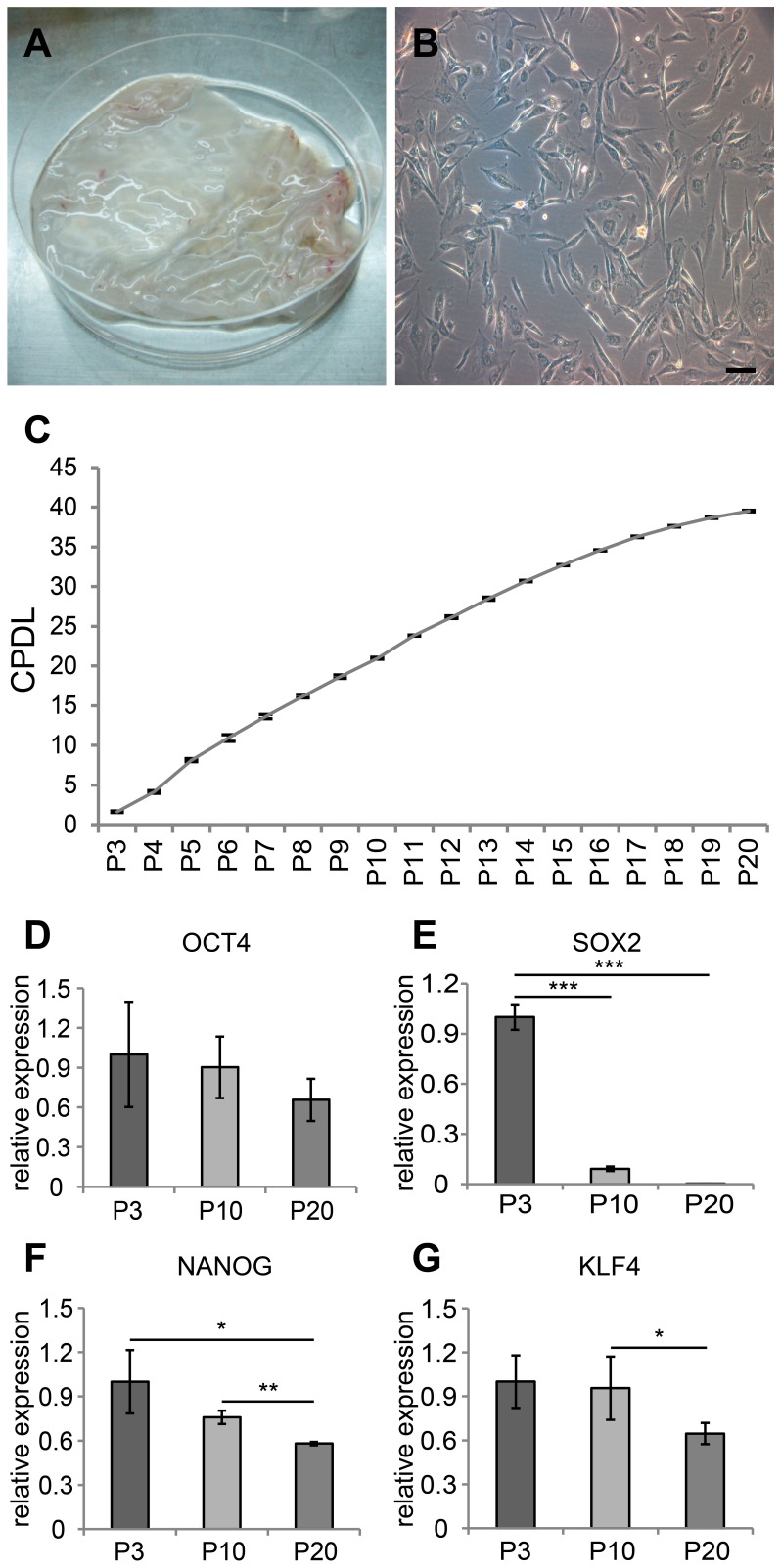
Primary culture of cAM-MSCs and the evaluation of CPDL & stem cell markers. (A) Harvesting of canine amniotic membrane tissue. (B) Phase contrast images of cAM-MSCs. Cells were cultured with DMEM (10% FBS). Cultured cells exhibited a fibroblast-like morphology and spindle shape similar to that displayed by human mesenchymal stem cells. Scale bar  = 50 µm. (C) Measuring CPDL of cAM-MSCs. CPDL was evaluated with the formula described in the Materials and Methods section. The CPDL was measured from passage 3 to 20. Cells grew consistently until passage 20. (D–G) Quantitative RT-PCR assay for evaluation of stem cell markers: OCT4, SOX2, NANOG and KLF4. We performed all these analyses in triplicate and the mean +/− the standard deviation plotted (*; p<0.5, **; p<0.01, ***; p<0.001).

### Adipogenesis

To determine if the cAM-MSCs could differentiate into adipocytes the cells were treated with adipogenic differentiation medium containing dexamethasone (1 uM), indomethacin (60 uM), 3-isobutyl-1-metyl-xanthine (500 uM; IBMX) and insulin (5 ug/ml) (Sigma-Aldrich, USA). The cells were treated with the adipogenic differentiation medium for 3 weeks at passage 5 once they had reached 80∼90% confluency. After 3 weeks, Oil Red O staining was performed to detect lipid droplets. The cells were fixed with 10% formalin for fixation at least 1 hour and rinsed with 60% isopropanol prior to incubation in freshly diluted Oil Red O for 10 minutes. Stains were solubilized using 100% isopropanol, and the resulting absorbande was measured at 500 nm using a spectrophotometer [11].

**Figure 2:**
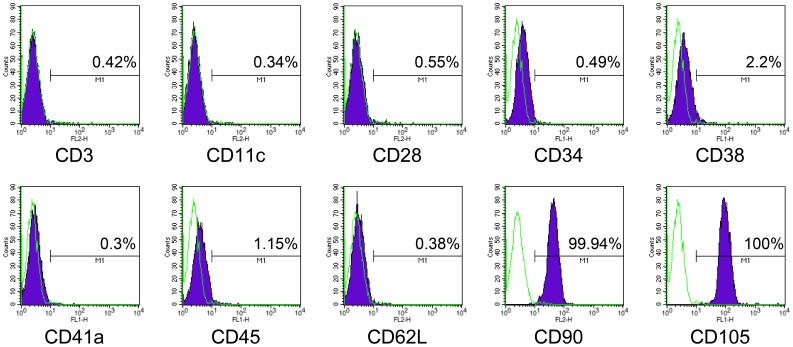
FACS analysis of cAM-MSCs. Analysis was performed at passage 5. Values show the intensity of the indicated antigen.

### Osteogenesis

Osteogenic differentiation medium containing ascorbic acid 2-phosphate (50 µM), dexamethasone (100 nM), β-glycerophosphate (10 mM) (Sigma-Aldrich, USA) and 10% FBS in LG-DMEM was used to determine osteogenic differentiation capability. When the cells reached at 80∼90% confluency, the medium was changed to the osteogenic differentiation medium, and the cells incubated for a further 3 weeks at passage 5. After 3 weeks, the calcium deposition was detected by staining with Alizarin Red S. The cells were washed with PBS and, fixation was performed with ice-cold ethanol (70%) for 1 hour at 4°C. The cells were then washed several times with distilled water. The cells were stained with Alizarin Red S (40 mM; pH 4.2; Sigma-Aldrich, USA) for 10 min at room temperature. The cells were rinsed of non-specific dye with five washes of distilled water. Alizarin Red S staining was solubilized using cetylpyridinium chloride (100 mM; Sigma-Aldrich, USA) for 1 hour. Solubilized Alizarin Red S absorbance was measured at 570 nm using a spectrophotometer [11].

**Figure 3:**
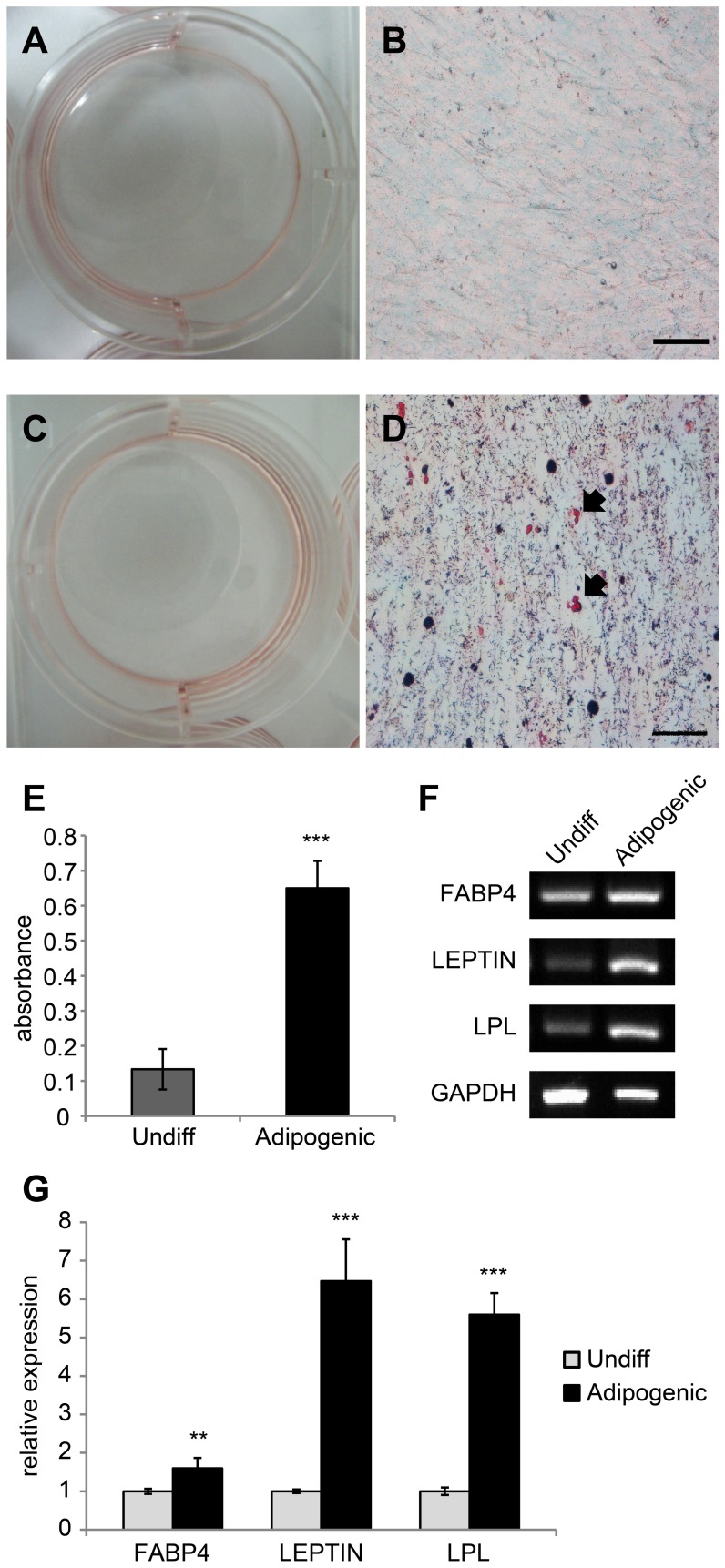
Adipogenic differentiation of cAM-MSCs. (A–D) Oil Red O staining after 3 weeks of adipogenic induction. (A, B) Control cells were grown in the basal culture medium. There was no staining with Oil Red O. (C, D) To assess adipogenic differentiation, the cells were treated with adipogenic induction medium. Fat droplets in differentiated cells were stained by Oil Red O. The black arrow indicates a stained red fat droplet. Scale bar  = 50 µm. For quantification, the stain was solubilized with 100% isopropanol, and absorbance was measured spectrophotometirically at 500 nm for 0.5 sec (E). Differentiated cells showed 5 fold greater values than control cells. We performed all these analyses in triplicate. (F, G) Gene expression levels were measured by RT-PCR (F) and quantitative RT-PCR (G) for adipogenic specific markers: FABP4, LEPTIN and LPL. GAPDH was used as a reference for evaluating the quality of mRNA. We performed all these analyses in triplicate and the mean +/− the standard deviation plotted (**; p<0.01, ***; p<0.001).

### Neurogenesis

Neural differentiation medium was used to induce neurogenesis. The cells were seeded at passage 5 in the basal culture medium and allowed to reach confluency. To induce differentiation, the cells were incubated with Beta-mercaptoethanol (1 mM; BME Sigma-Aldrich, USA) and 5% FBS for 24 hours prior to induction. Following this, the cells were treated with serum-free induction medium containing Docosahexaenoic (100 µM; DHA, Sigma-Aldrich, USA), B27 supplement (Gibco BRL, USA) and 1.5% Dimethyl sulfoxide (DMSO, Sigma-Aldrich, USA for 2 days [12].

### Chondrogenesis

To promote the cAM-MSCs differentiate into chondrocytes, chondrogenic differentiation medium was used. The cells (5×10^5^) were seeded in a 15 ml polypropylene tube and centrifuged to a pellet at passage 5. The pellets were cultured in 1 ml of chondrogenic differentiation medium (Lonza) and incubated for 3 weeks. The medium was changed 3 times a week. After differentiation, the pellets were embedded in paraffin and 3 µm sections were cut. To detect chondrogenesis, the sections were stained with toluidine blue following standard protocols [13].

**Figure 4:**
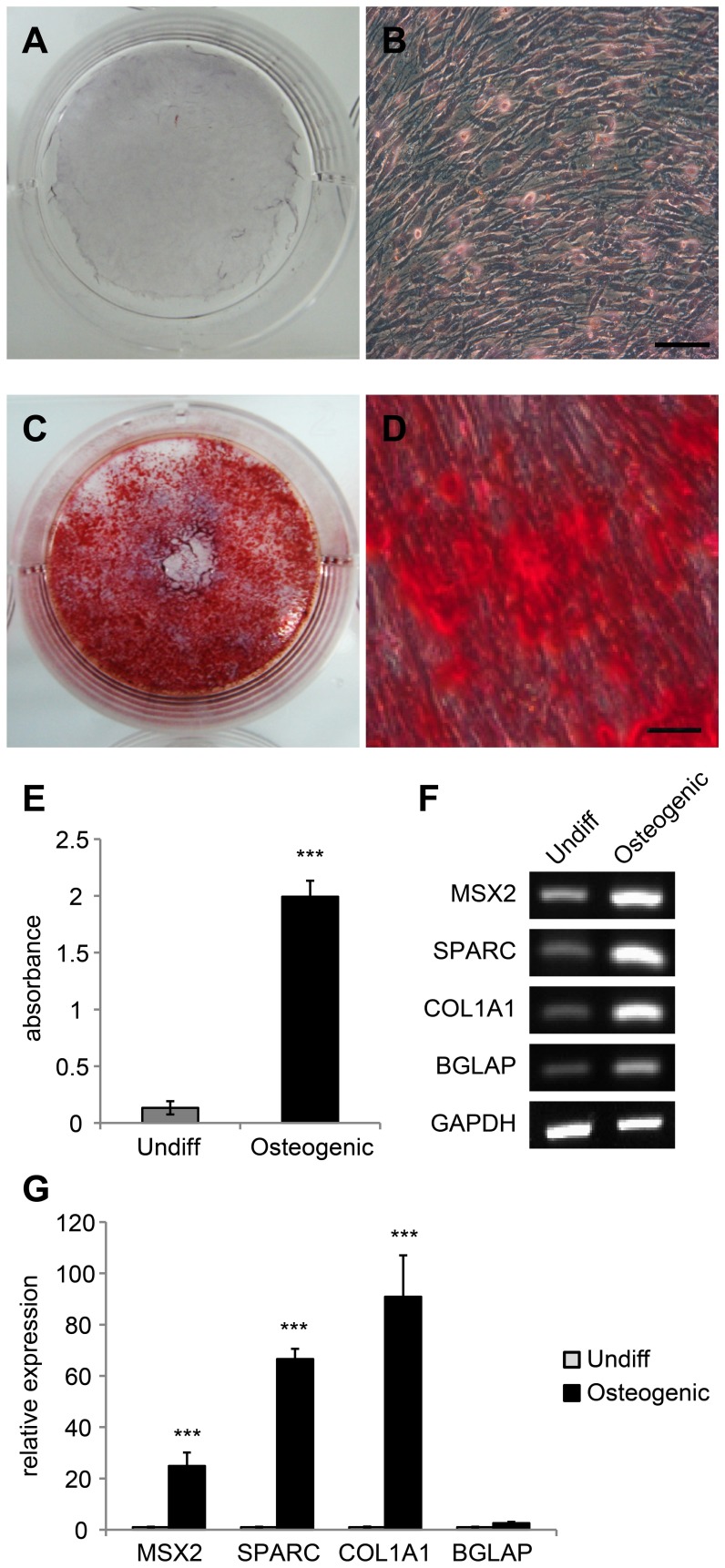
Osteogenic differentiation of cAM -MSCs. (A–D) Cultured were stained with Alizarin Red S after 3 weeks to detect osteogenic induction. (A, B) Control cells were grown in basal culture medium. No staining with Alizarin Red S was observed. (C, D) Cells grown in osteogenic induction medium stained strongly with Alizarin Red S, compared to control cells. Scale bar  = 50 µm. For quantification, stains were solubilized with 100 mM cetylpyridinium chloride, and the absorbance was measured spectrophotometrically at 570 nm for 0.5 sec (E). Differentiated cells showed 15-fold greater values than control cells. We performed all these analyses in triplicate. (F, G) Gene expression levels were measured by RT-PCR (F) and quantitative RT-PCR (G) for osteogenic specific markers: MSX2, SPARC, COL1A1 and BGLAP. GAPDH was used as a reference for evaluating the quality of mRNA. We performed all these analyses in triplicate and the mean +/− the standard deviation plotted (***; p<0.001).

### Immunostaining

Mouse anti-neural specific beta III tubulin (Abcam, UK) and rabbit anti-Glial Fibrillary Acidic Protein (GFAP, Millipore, USA) antibodies were used for immunostaining. Cells were fixed with 4% paraformaldehyde for 20 min, and were then permeabilized in 0.5% Triton-X 100 at room temperature for 10 min. After washing with PBS several times, the cells were blocked with 10% normal goat serum (NGS) overnight at 4°C. Primary antibodies were incubated for 2 hours at room temperature. After PBS washing, the cells were incubated with secondary antibodies Alexa 488 & 594 (1∶1000, Molecular Probe, Inc., Eugene, OR, USA) for 1 hour. Finally, for nuclear staining, the samples were incubated for 15 minutes with Hoechst 33238 (1 mg/ml), diluted 1∶100 in PBS. Images were captured using a confocal microscope (Eclipse TE2000; Nikon, Japan).

**Figure 5:**
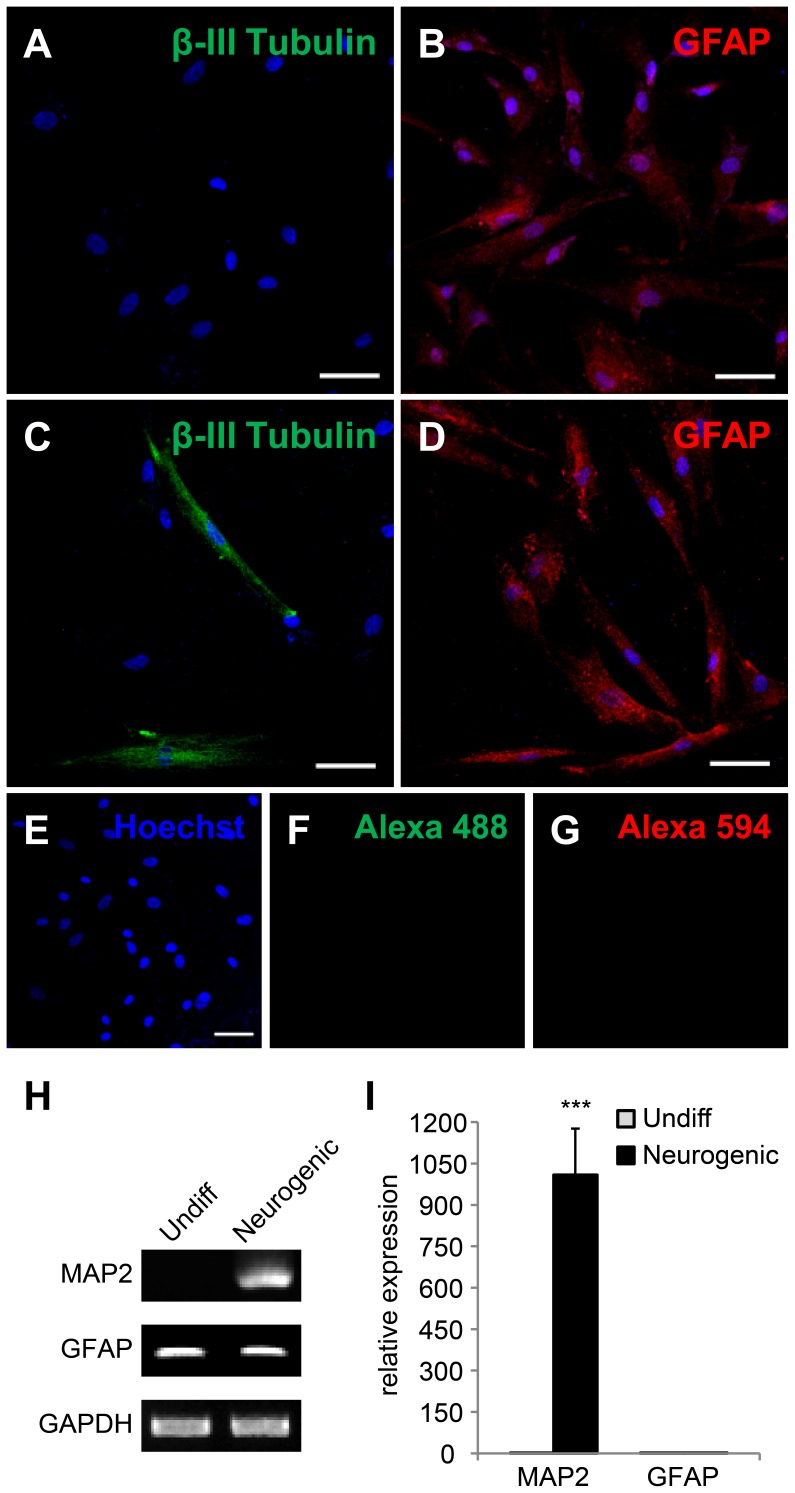
Neural differentiation of cAM-MSCs. The cells were immunostained with GFAP and beta III tubulin, neural specific markers. (A, B) Control cells were cultured with the basal culture medium. Control cells were positive for GFAP but not for beta III tubulin. (C, D) After neural differentiation, cells were stained with GFAP and beta III tubulin. Scale bar = 50 µm. (E–G) Negative controls were performed only with Alexa 488 (green, F), Alexa 594(red, G) and Hoechst staining for nuclei detection (blue, E). (H, I) Gene expression levels were measured by RT-PCR (H) and quantitative RT-PCR (I) for neural specific markers: MAP2 and GFAP. GAPDH was used as a reference for evaluating the quality of mRNA. We performed all these analyses in triplicate and the mean +/− the standard deviation plotted (***; p<0.001).

### RT-PCR

Total RNA was extracted from the cultured cells with the Easy-spin total RNA extraction kit (Intron Biotechnology, Seongnam, Korea) according to the manufacturer’s protocol. RNA concentrations were measured by absorbance at 260 nm with a spectrophotometer. cDNA was prepared by 1 µg of total RNA for reverse transcription using Superscript II reverse transcriptase (Invitrogen, Carlsbad, CA) and oligo (dT) primers (Invitrogen). The cDNA was amplified by PCR using Platinum Taq (Invitrogen, Carlsbad, CA). The PCR primers are shown in the Table 1. The PCR products were separated on a 1.5% agarose gel and visualized with ethidium bromide.

### Quantitative RT-PCR

Quantitative RT-PCR was performed by mixing cDNA with primers and Power SYBR Green PCR Master Mix (Applied Biosystems, Foster City, CA). Quantitative RT-PCR was performed using an ABI 7500 Realtime-PCR System with supplied software (Applied Biosystems), according to the manufacturer's instructions. RNA expression levels were compared after normalization to endogenous glyceraldehyde-3-phosphate dehydrogenase (GAPDH). The primer sequences used in this study are listed in Table 1 & 2.

## Results

### Primary Culture of cAM-MSCs

Placental tissues were collected from canine cesarean-section deliveries. We mechanically peeled off the amniotic membrane tissues from the placental tissue (Figure 1A). We isolated and cultured the primary stem cells from the canine amniotic membrane tissue. The cAM-MSCs displayed the spindle-shaped morphology typical of MSCs, and were adherent to the plastic culture surface (Figure 1B). For cell proliferation assays, we measured and calculated the cell population via CPDL. The cells were seeded (5×10^4^ cells/well) in a 6-well culture plate and subcultured 5–7 days later. This was repeated until we observed a decrease in the proliferation rate, from passage 3 to 20. The curve of the rate of growth steadily increased through the cumulative population was observed (Figure 1C).

**Figure 6:**
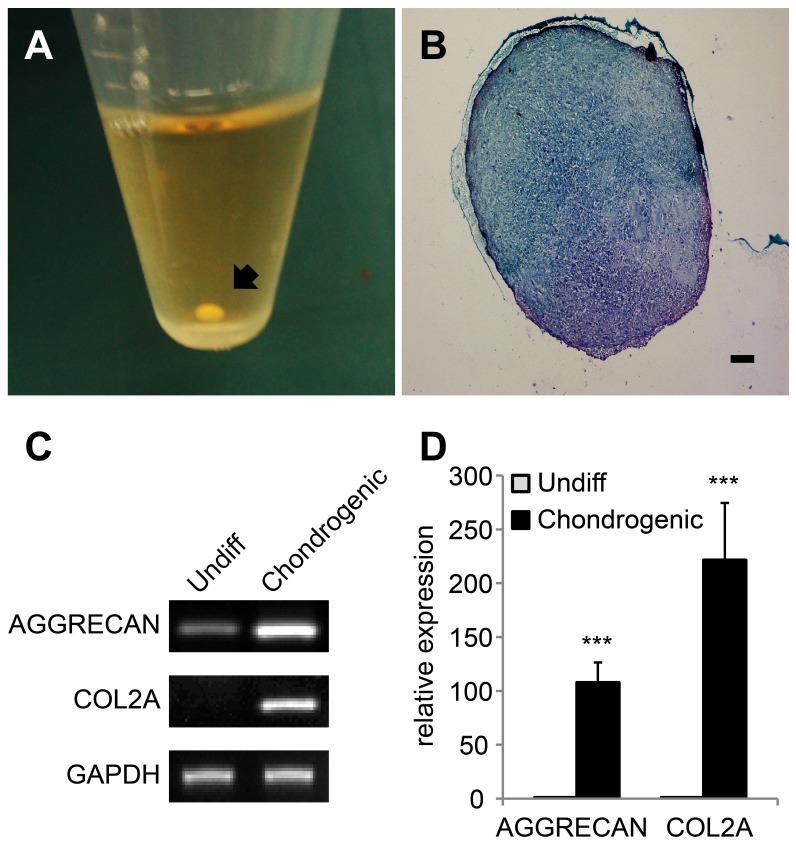
Chondrogenic differentiation of cAM-MSCs. After 3 weeks of chondrogenic induction, pellet formation was observed. (A) Image of an ovoid-shaped chondrogenic pellet. The pellet was formed at the bottom of a 15 ml polypropylene tube. The black arrow indicates a pellet. (B) Toluidine blue staining of chondrogenic pellets. The pellets were embedded in paraffin and cut into 3 µm slices, which were mounted on slides. Slides were stained with toluidine blue. The stained tissue showed a typical, cartilaginous tissue phenotype. Scale bar  = 100 µm. (C, D) Gene expression levels were measured by RT-PCR (C) and quantitative RT-PCR (D) for chondrogenic specific markers AGGRECAN and COL2A. GAPDH was used as a reference for evaluating the quality of mRNA. We performed all these analyses in triplicate and the mean +/− the standard deviation plotted (***; p<0.001).

### Expression Pattern of Stem Cell Markers

To measure gene expression levels of stem cell markers, quantitative RT-PCR was performed in passage dependant manner (passage 3, 10 and 20). The stem cell markers, such as OCT4, SOX2, NANOG and KLF4, showed the decreasing expression pattern as a function of increased passages (Figure1 D–G).

### Immunophenotype of cAM-MSCs

To identify the immunophenotype of cAM-MSCs, cell surface specific markers were examined via FACS analysis. Commonly, MSCs show definite specific cell surface markers. According to the International Society of Cellular Therapy, MSCs have positive expression of CD90 and CD105, but negative expression of CD11c, CD34 and CD45 surface antigens [14]. For FACS analysis of cAM-MSCs, we used 10 CD markers (CD3, CD11c, CD28, CD34, CD38, CD41a, CD45, CD62L, CD90 and CD105) to distinguish the MSC phenotype. For FACS analysis showed that, cAM-MSCs have an expression pattern consistent with the MSC immunophenotype (Figure 2). The results showed that cAM-MSCs showed positive expression of CD90 and CD105, which are well-known, typical MSCs markers. CD90 is called Thy-1 and is a marker for various types of stem cells, such as endometrial stem cells, hepatic stem cells, keratinocyte stem cells and mesenchymal stem cells [15]–[17]. CD105 is also called SH2 and is a well-known MSC marker [14]. However, the cells were negative for the expression of other immune cells markers (CD3, CD11c, CD28, CD38 and CD62L), hematopoietic cells (CD34, CD45) and markers of platelets (CD41a). These results show that the immunophenotype of the cAM-MSCs is consistent with that of other characterized MSCs.

### Adipogenesis

To confirm their adipogenic differentiation ability, cAM-MSCs were treated with adipogenic induction medium for 3 weeks. After differentiation, the cells were stained with Oil Red O for the detection of fatty droplets. The basal culture medium was used as a control condition. We detected the fatty droplets under differentiation conditions, but not under control conditions (Figure 3A–D). To quantify the differentiation status of the cells, the Oil Red O was eluted with 100% isopropanol and the absorbance measured (Figure 3E). The differentiated cells displayed absorbance values that were 5-fold greater than those of the control cells. Additionally, we measured the gene expression levels of markers associated with adipogenesis, such as FABP4, Leptin and LPL via RT-PCR (Figure 3F) and quantitative RT-PCR (Figure 3G). After differentiation, the adipogenic-associated markers were increased in treated cells compared to controls (Figure 3F, G).

### Osteogenesis

To confirm their osteogenic differentiation ability, cAM-MSCs were treated with osteogenic induction medium for 3 weeks. Alizarin Red S staining, which positively stains calcium depositions, was used to detect differentiation. Basal culture medium was used as a control condition. Under differentiation conditions, there was strong, positive Alizarin Red S staining. Negative staining was observed under control conditions (Figure 4A–D). To quantify the differentiation status, stain from all cells was eluted with 100 mM of cetylpyridinium chloride and absorbance measured. The differentiated cells displayed about 15-fold greater values than control cells. Additionally, we measured the gene expression levels of markers associated with osteogenesis, such as MSX2, SPARC, COL1A1 and BGLAP via RT-PCR (Figure 4F) and quantitative RT-PCR (Figure 4G). After differentiation, the osteogenic associated markers were increased compared to controls (Figure 4F, G).

### Neurogenesis

To confirm their differentiation ability, cAM-MSCs were treated with neural differentiation medium. After differentiation, we performed immunostaining and RT-PCR. Basal culture medium was used as a control condition. By immunostaining, we showed that neural markers GFAP and beta III tubulin were positively expressed under differentiation conditions (Figure 5C, D). However, under the basal culture condition, cAM-MSCs expressed GFAP but, not beta III tubulin (Figure 5A, B). The negative control was incubated with secondary antibodies Alexa 488 & 594 demonstrated no background signal (Figure 5E–5F). When we measured the expression levels of neural-associated genes via RT-PCR (Figure 5H) and quantitative RT-PCR (Figure 5I), we found that GFAP was expressed under both control and neural differentiation conditions. Under differentiation conditions, MAP2 expression was positive compared to control conditions (Figure 5H, I).

### Chondrogenesis

To confirm their chondrogenic differentiation ability, cAM-MSCs were cultured with the chondrogenic differentiation medium. The cells were seeded into 15 mL polypropylene tubes and centrifuged to from a pellet. The cell pellets were incubated with the differentiation medium for 3 weeks. We found that the pellets formed in the bottom of polypropylene tube had an ovoid shape and an opaque body (Figure 6A). Toluidine blue staining was performed to identify chondrogenesis. After differentiation, the pellet showed positive toluidine blue staining (Figure 6B). We also measured the expression patterns of genes associated with chondrogenic markers, such as Aggrecan and COL2A1 via RT-PCR (Figure 6C) and quantitiative RT-PCR (Figure 6D). Chondrogenic markers were increased under differentiation conditions, compared to control conditions (Figure 6C, D).

## Discussion

The placenta consists of tissue both maternal and fetal origin after implantation. The placenta consists of fetal placenta from the fetus and uterine placenta from the mother, which is called placentation. The placenta is comprised three-layer structure of the amnion, chorion and decidua. The roles of the placenta are to provide nutrients and oxygen, which are essential for fetal survival and development. The amnion is a thin, nonvascular membrane, which has a two-layer structure: an epithelial monolayer and a stromal layer [18]. In particular, the amnion is the sac that binds the fetus and constructs the environment. The parturient placenta was previously discarded and classified as medical wastes, however, the amniotic membrane has clinical applications in covering wounds and burn lesions and ocular surface reconstruction [7]. In humans, some reports have demonstrated the isolation and characterization of mesenchymal stem cells from whole placenta and amniotic membrane [7]–[9]. Significantly, they have reported the identification of epithelial stem cells from the amniotic membrane [19]. These isolated cells have the characteristics of stem cells, including, self-renewal and multi-lineage differentiation abilities. Placental derived-stem cells are useful tools for regenerative medicine. They have advantages in cell applications, including the immune privilege, the lack of ethical issues and non-invasive procedures to obtain the amnion. Recently, canine stem cells have been studied for use in cell therapy [20], [21]. However, they have been confined to limited stem cell sources. Most of the cells were isolated from canine adipose-derived tissue, umbilical cord blood or bone marrow [13], [22], [23]. Therefore, an established diversity of stem cell source is needed.

In our study, we isolated and cultured cells from 6 different canine amniotic membrane samples (the rate of success was 100%). All isolated cells (from 6 samples) showed a very similar cell morphology and ability to be subcultured. We randomly selected three cell lines for characterization (line2 and line3 were described in supporting data; Figure S1, S2, S3, S4, and S5 and Table S1). All experiments, CPDL, FACS analysis and differentiation studies, were conducted by only the selected cell line (in triplicate).

In our study, we showed the isolation and characterization of MSCs from canine amniotic membranes. Also, Uranio et al. [24] published the study for isolation of canine MSCs from amniotic membranes. However, the article has lack of detailed isolation method and important stem cell characteristics, such as long-term cell maintenance, the immunophenotype, the comprehensive confirmation in differentiation studies. These researches are important for isolation & characterization of stem cells and veterinary medicine.

The amniotic membrane was collected from the subjacent chorion by mechanical detachment. The amniotic membrane was digested with an enzyme treatment for cell isolation. After digestion, cAM-MSCs were grown in basal cultured medium (LG-DMEM with 10% FBS) for 20 passages. To identify the characteristics of stem cells, we assayed their self-renewal and differentiation abilities. The cell proliferation rate of cAM-MSCs was evaluated via the calculation of cumulative population doubling levels from passage 3 to 20. The stem cell markers were confirmed in passage dependant manner. The morphology of cAM-MSCs was similar to that of typical MSCs with a spindle, fibroblast-like-shape. They also displayed adherence to the plastic culture surface [25]. Immunophenotyping of cAM-MSCs was performed by FACS analysis. The cells expressed the mesenchymal stem cells markers CD90 and CD105, but not hematopoietic surface markers such as CD34 and CD45. The expression pattern of markers indicated that cAM-MSCs have an expression pattern that is characteristic of MSCs [22]. Immnuophenotype characterization indicated that cAM-MSCs are positive for the mesenchymal stem cells markers, CD90 (99.94%) and CD105 (100%). In hematopoietic surface makers, there showed negative or some expression pattern for CD45 (1.15%) and CD34 (0.49%). In the case of MSCs, usually, CD34 has a negative expression, but Viera et al. [22] and Oh et al. [26] showed that CD34-positive cells (7–10%) in canine adipose-derived mesenchymal stem cells are more positive population than ours. However, in case of other stem sources, such as bone marrow, amniotic fluid, amnion and umbilical cord matrix, showed negative population of CD34 in MSCs [24], [27]. In this regard, we expect that the population of CD34 cells has some differences from origins of stem cells in canine.

We observed the multipotent differentiation ability of cAM-MSCs under various induction conditions. We carried out differentiation studies of osteogenesis, adipogenesis, neurogenesis and chondrogenesis. In the adipogenesis differentiation sutdy, we confirmed that cAM-MSCs displayed fatty droplets, which were positively stained with Oil Red O under adipogenic induction conditions. We then eluted the staining in order to quantify differentiation levels, compared to control conditions. By measuring gene expression levels, we showed that adipogenic associated markers were increased after the induction of adipogenic differentiation. After induction of osteogenic differentiation, cAM-MSCs were positively stained with Alizarin Red S. The level of staining was quantified by elution. We showed that the expression levels of osteogenic marker genes, were increased after differentiation. In the neurogenesis differentiation study, we measured the expression of neural-associated markers by immunostaining and RT-PCR. cAM-MSCs express GFAP, as shown by the measurement of both protein and gene levels, even under basal non-differentiating conditions. We confirmed that neural specific markers beta III tubulin and MAP2 were expressed under differentiation conditions. We also found that cAM-MSCs could be differentiated into chondrogenic lineage cells. We confirmed that cAM-MSCs formed pellets, which were positive for toluidine Blue staining under chondrogenic induction medium conditions. Our RT-PCR results showed increased gene levels of markers associated with chondrogenesis.

Taken together, these results show that we have isolated stem cells from amniotic membrane, which have both the characteristics of MSCs and self-renewal ability. Therefore, cAM-MSCs could be a useful source of stem cells for the research of stem cells in canine. Additionally, we suggest that cAM-MSCs have potential therapeutic properties for use in regenerative medicine.

## Supporting Information

Figure S1 **Primary culture and the evaluation of CPDL.** (A, B) Harvesting of canine amniotic membrane tissue. (C, D) Phase contrast images of cAM-MSCs. Scale bar = 50 µm. (E) Measuring CPDL of cAM-MSCs. CPDL was evaluated with the formula described in the Materials and Methods section. The CPDL was measured from passage 3 to 20. Cells grew consistently until passage 20.
(JPG)

Figure S2 **Adipogenic differentiation.** (A–D) Oil Red O staining after 3 weeks of adipogenic induction. (A, C) Control cells were grown in the basal culture medium. There was no staining with Oil Red O. (B, D) To assess adipogenic differentiation, the cells were treated with adipogenic induction medium. Fat droplets in differentiated cells were stained by Oil Red O. Scale bar  = 50 µm. (E, F) For quantification, the stain was solubilized with 100% isopropanol, and absorbance was measured spectrophotometirically at 500 nm for 0.5 sec. We performed all these analyses in triplicate and the mean +/− the standard deviation plotted (***; p<0.001). (G, H) Gene expression levels were measured by RT-PCR.
(JPG)

Figure S3 **Osteogenic differentiation.** (A–D) The cells were stained with Alizarin Red S after 3 weeks to detect osteogenic induction. (A, C) Control cells were grown in basal culture medium. No staining with Alizarin Red S was observed. (B, D) Cells grown in osteogenic induction medium stained strongly with Alizarin Red S, compared to control cells. Scale bar = 50 µm. (E, F) For quantification, stains were solubilized with 100 mM cetylpyridinium chloride, and the absorbance was measured spectrophotometrically at 570 nm for 0.5 sec. We performed all these analyses in triplicate and the mean +/− the standard deviation plotted (***; p<0.001). (G, H) Gene expression levels were measured by RT-PCR.
(JPG)

Figure S4 **Neural differentiation.** (A, B) Gene expression levels were measured by RT-PCR.
(JPG)

Figure S5 **Chondrogenic differentiation.** (A, D) Image of an ovoid-shaped chondrogenic pellet. The pellet was formed at the bottom of a 15 ml polypropylene tube. (B, E) Toluidine blue staining of chondrogenic pellets. Scale bar = 100 µm. (C, F) Gene expression levels were measured by RT-PCR.
(JPG)

Table S1 **Expression patterns of CD markers with Cell line-2 and Cell line-3.** The Values were measured by percentage.
(DOCX)
