# Supplementary figures and images for: Isolation and Characterization of Canine Amniotic Membrane-Derived Multipotent Stem Cells
Source: PLoS One. 2012 Sep 14;7(9):e44693. doi: 10.1371/journal.pone.0044693 (PMC3443096; doi:10.1371/journal.pone.0044693)

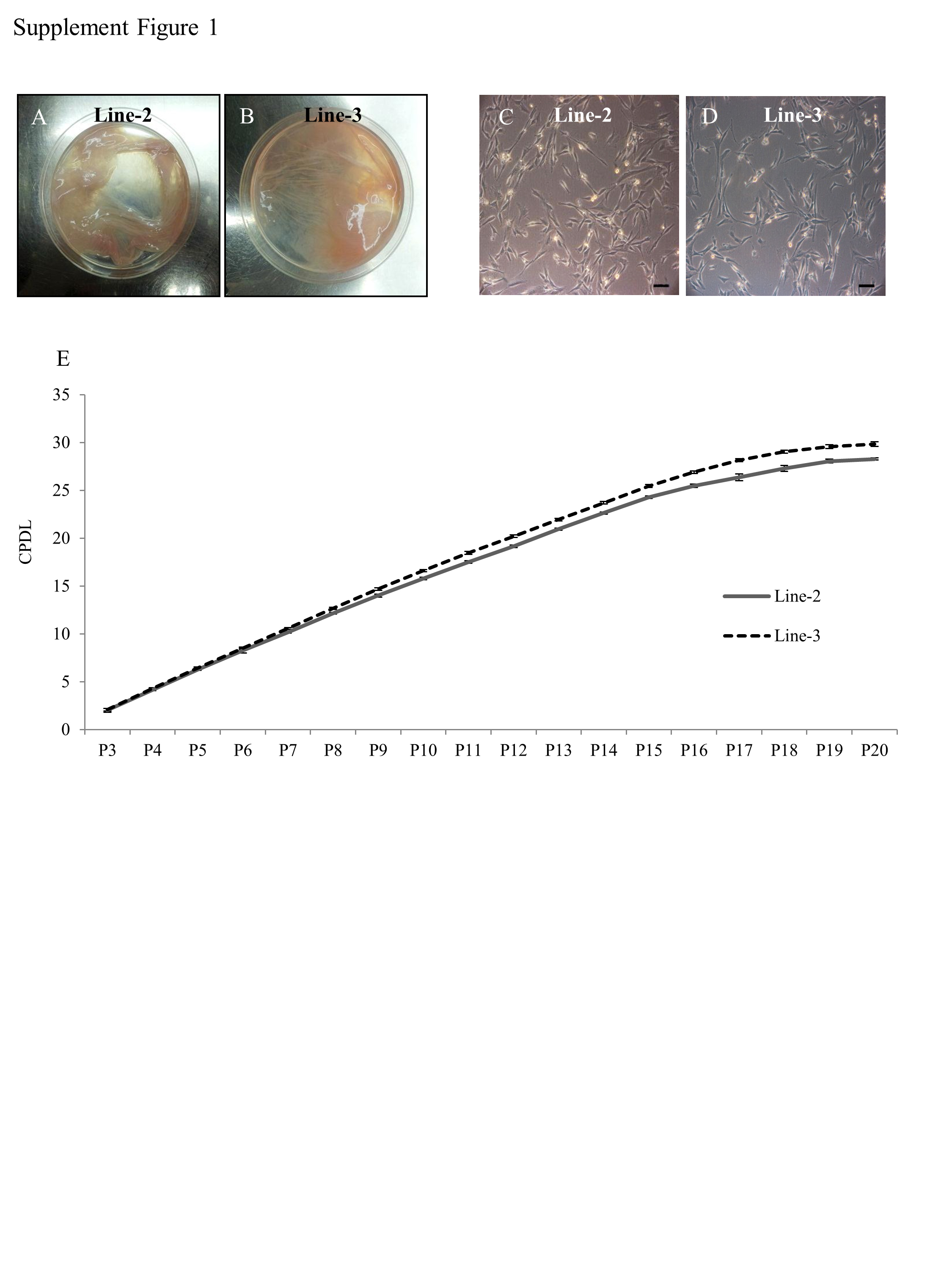

Supplement: Figure S1 — Primary culture and the evaluation of CPDL. (A, B) Harvesting of canine amniotic membrane tissue. (C, D) Phase contrast images of cAM-MSCs. Scale bar = 50 µm. (E) Measuring CPDL of cAM-MSCs. CPDL was evaluated with the formula described in the Materials and Methods section. The CPDL was measured from passage 3 to 20. Cells grew consistently until passage 20. (JPG) [file pone.0044693.s001.jpg]

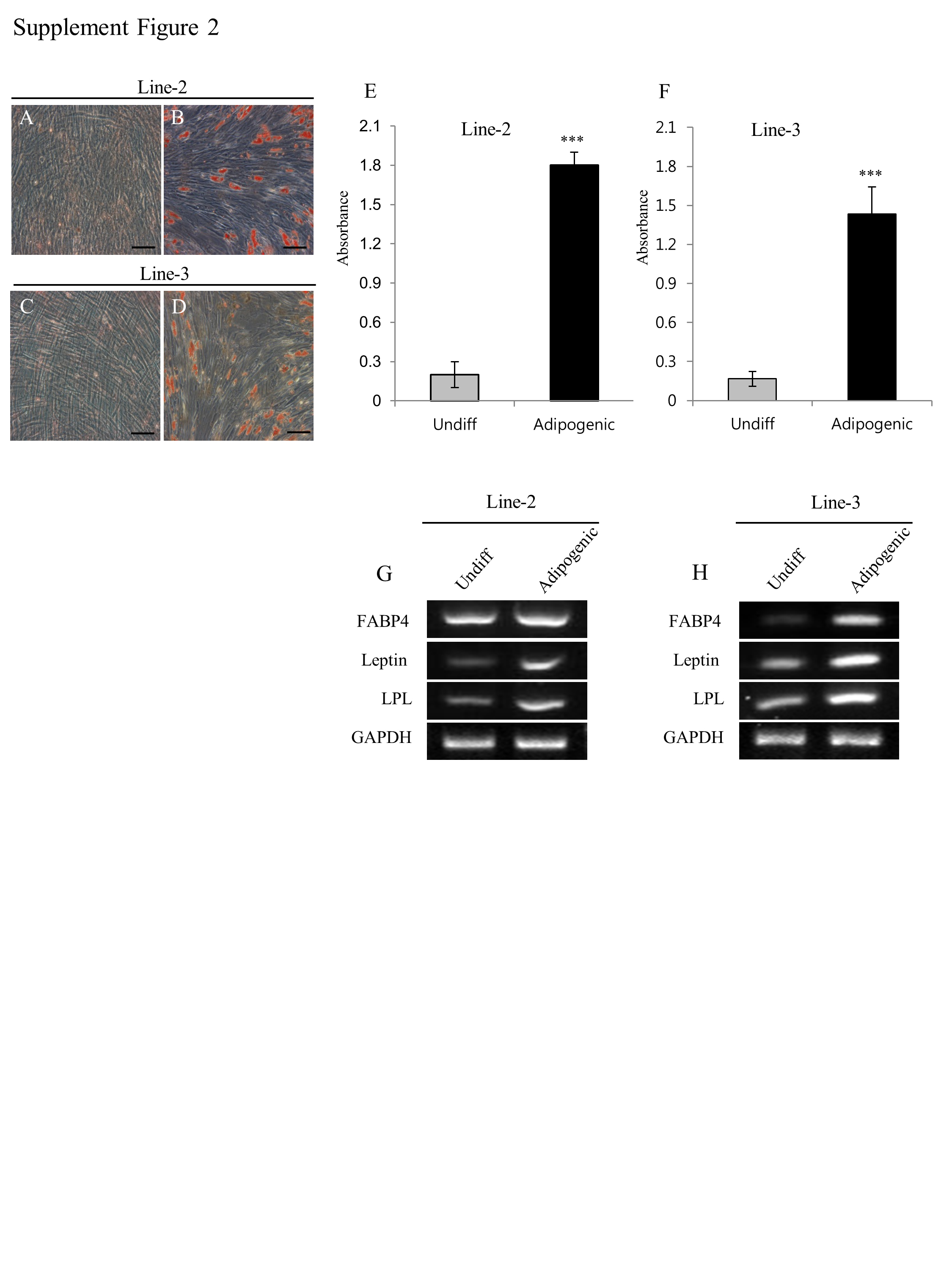

Supplement: Figure S2 — Adipogenic differentiation. (A–D) Oil Red O staining after 3 weeks of adipogenic induction. (A, C) Control cells were grown in the basal culture medium. There was no staining with Oil Red O. (B, D) To assess adipogenic differentiation, the cells were treated with adipogenic induction medium. Fat droplets in differentiated cells were stained by Oil Red O. Scale bar = 50 µm. (E, F) For quantification, the stain was solubilized with 100% isopropanol, and absorbance was measured spectrophotometirically at 500 nm for 0.5 sec. We performed all these analyses in triplicate and the mean +/− the standard deviation plotted (***; p<0.001). (G, H) Gene expression levels were measured by RT-PCR. (JPG) [file pone.0044693.s002.jpg]

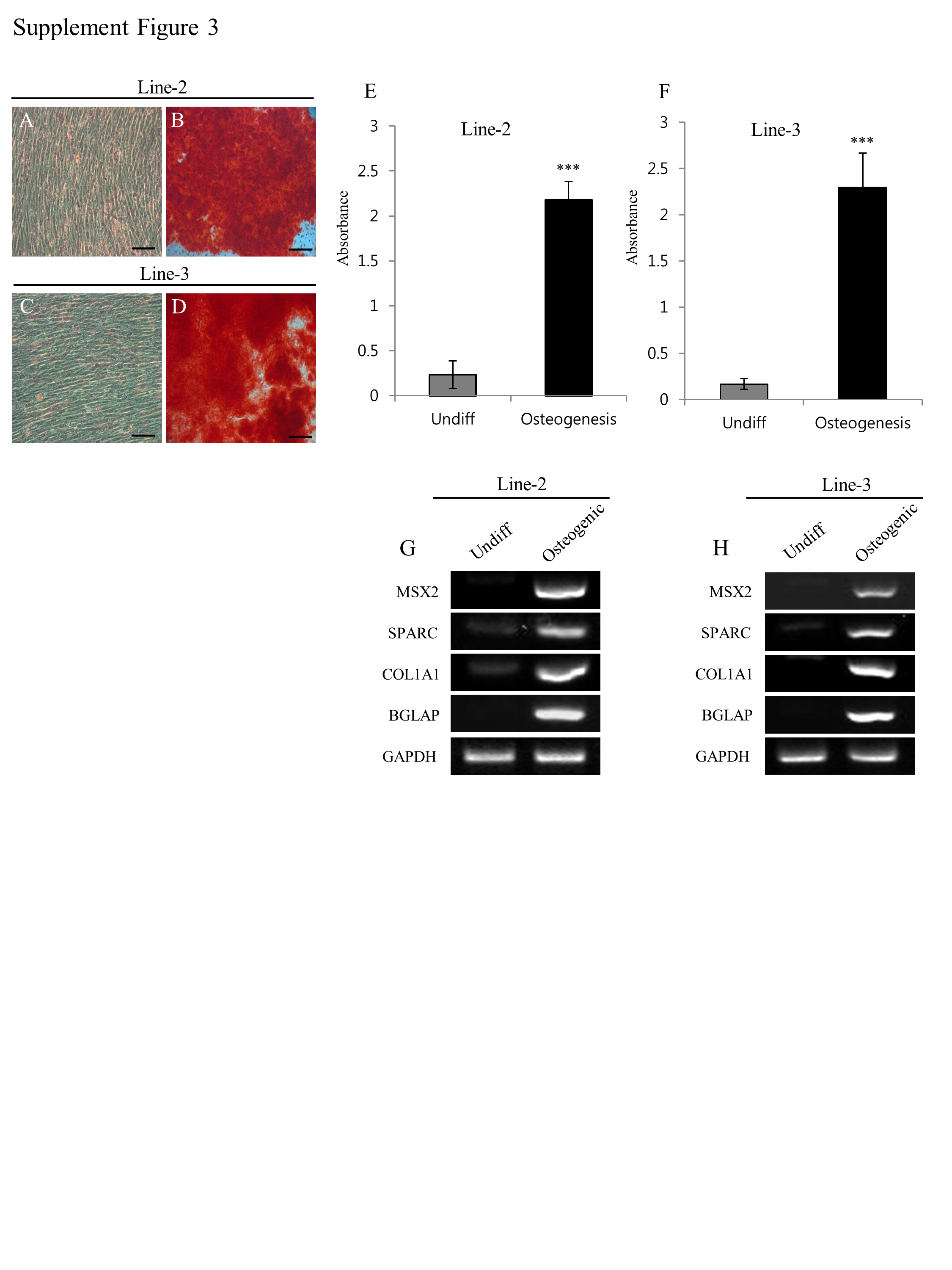

Supplement: Figure S3 — Osteogenic differentiation. (A–D) The cells were stained with Alizarin Red S after 3 weeks to detect osteogenic induction. (A, C) Control cells were grown in basal culture medium. No staining with Alizarin Red S was observed. (B, D) Cells grown in osteogenic induction medium stained strongly with Alizarin Red S, compared to control cells. Scale bar = 50 µm. (E, F) For quantification, stains were solubilized with 100 mM cetylpyridinium chloride, and the absorbance was measured spectrophotometrically at 570 nm for 0.5 sec. We performed all these analyses in triplicate and the mean +/− the standard deviation plotted (***; p<0.001). (G, H) Gene expression levels were measured by RT-PCR. (JPG) [file pone.0044693.s003.jpg]

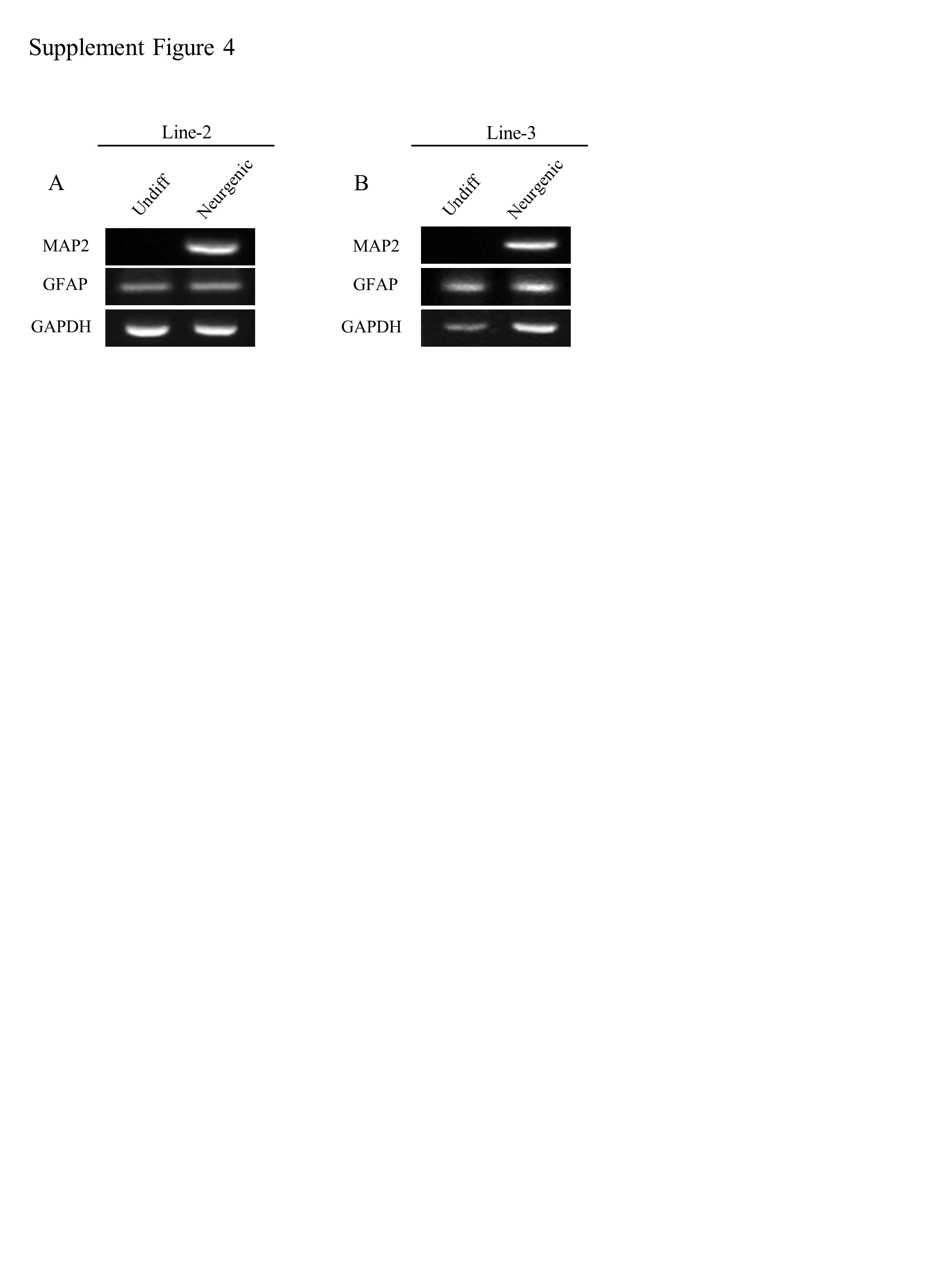

Supplement: Figure S4 — Neural differentiation. (A, B) Gene expression levels were measured by RT-PCR. (JPG) [file pone.0044693.s004.jpg]

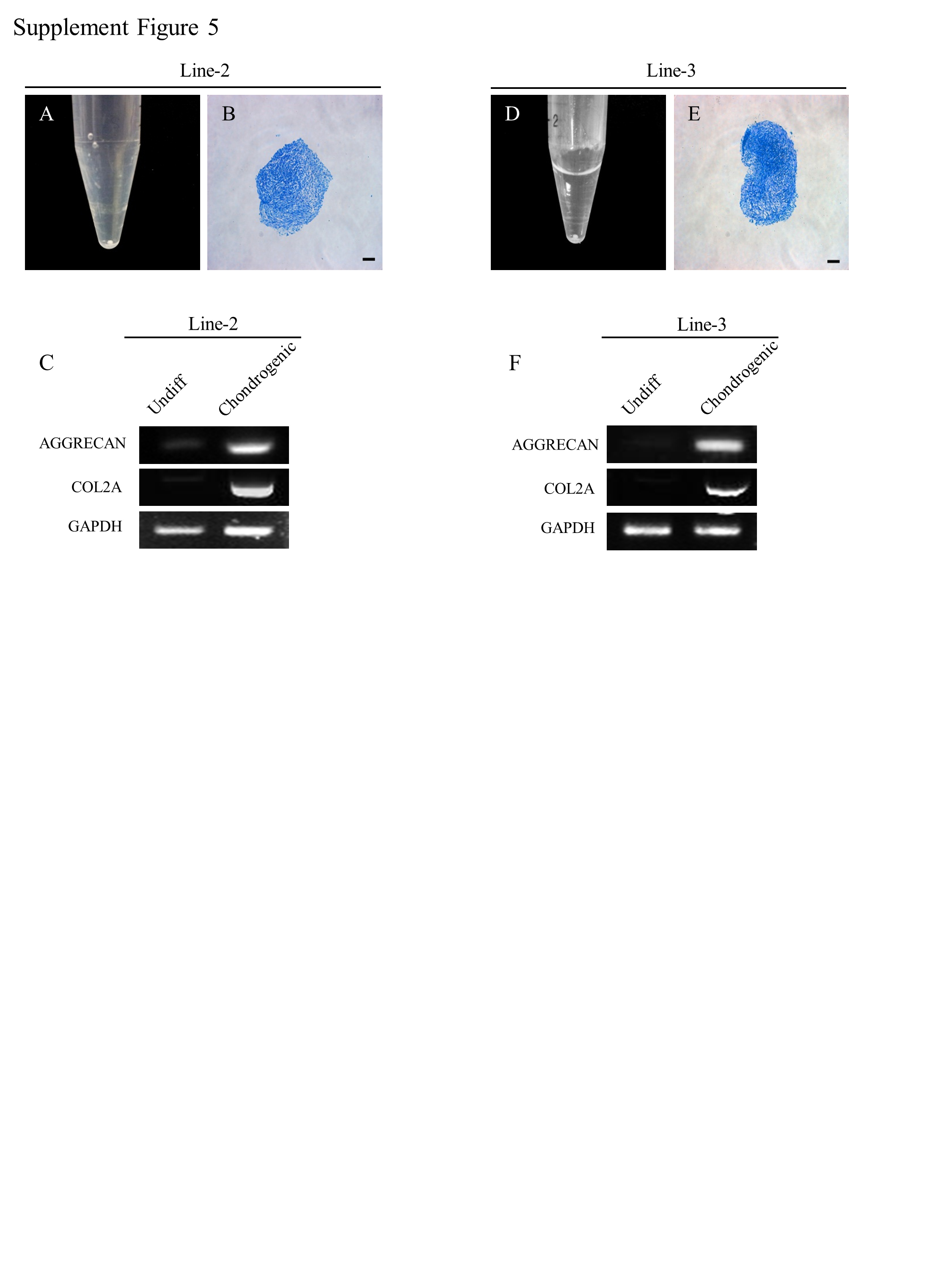

Supplement: Figure S5 — Chondrogenic differentiation. (A, D) Image of an ovoid-shaped chondrogenic pellet. The pellet was formed at the bottom of a 15 ml polypropylene tube. (B, E) Toluidine blue staining of chondrogenic pellets. Scale bar = 100 µm. (C, F) Gene expression levels were measured by RT-PCR. (JPG) [file pone.0044693.s005.jpg]
