# Supplementary material for: Isolation and Characterization of Canine Amniotic Membrane-Derived Multipotent Stem Cells
Source: PLoS One. 2012 Sep 14;7(9):e44693. doi: 10.1371/journal.pone.0044693 (PMC3443096; doi:10.1371/journal.pone.0044693)
Supplement: Table S1 — Expression patterns of CD markers with Cell line-2 and Cell line-3. The Values were measured by percentage. (DOCX) [file pone.0044693.s006.docx]

**Supporting Table**

Table S1.

| CD markers | Cell Line-2 | Cell Line-3 |
| --- | --- | --- |
| CD3 | 1.28% | 0.42% |
| CD11c | 0.96% | 0.8% |
| CD28 | 1.25% | 0.35% |
| CD34 | 2.29% | 0.41% |
| CD38 | 0.81% | 0.56% |
| CD41a | 1.23% | 1.84% |
| CD45 | 0.6% | 0.46% |
| CD62L | 0.93% | 0.41% |
| CD90 | 100% | 100% |
| CD105 | 99.85% | 99.48% |
